# Enhanced production of xylanase by solid state fermentation using *Trichoderma koeningi* isolate: effect of pretreated agro-residues

**DOI:** 10.1007/s13205-014-0239-4

**Published:** 2014-07-30

**Authors:** Ramesh Bandikari, Vijayakumar Poondla, Vijaya Sarathi Reddy Obulam

**Affiliations:** Department of Biochemistry, Sri Venkateswara University, Tirupati, 517 502 India

**Keywords:** Agro**-**residues, Pretreatment, SSF, Xylanase, Xylose, Xylo**-**oligosaccharides

## Abstract

The main objective of this study was to isolate the fungal strain for enhanced production of xylanase using different agro**-**residues and fruit peels by solid state fermentation and its potentiality was tested on the pretreated corn cob. Fermentation was carried out with *Trichoderma koeningi* isolate using untreated and pretreated corn cob supplemented with pineapple peel powder showed higher production of xylanase 2,869.8 ± 0.4 (IU/g) and extracellular protein 7.6 ± 0.2 (mg/g) of corn cob, in the latter than the former yielding 1,347.2 ± 0.7 (IU/g) and 4.9 ± 0.1 (mg/g) of corn cob, respectively, at pH 6.5 and incubation period for 96 h. In the FT-IR spectrum, the bands at 1,155, 1,252 and 1,738 cm^−1^ had disappeared. This indicates the depolymerization of hemicellulose and the band at 1,053 cm^−1^ shows the presence of β (1-4)-xylan in the pretreated corn cobs. The pretreated biomass hydrolysed with a xylanase concentration of 14 U and 6 h incubation showed mainly xylose and its oligosaccharides, which were quantified using HPLC. From the results we can conclude that pretreated energy**-**value and cheaply available agro-residues can be effectively used as substrates for the enhanced production of xylanase.

## Introduction

Xylan is the second abundant polysaccharide after cellulose in plant cell walls and its structure is composed of β-1,4-linked-d-xylopyranose (heterogeneous) residues. It comprises 20–35 % dry weight of wood and agricultural wastes and is the major component of the hemicellulose portion. The industrial enzymes production is often limited by the cost of substrates for the cultivation of the microorganisms. The use of low cost substrates, such as agricultural wastes has been suggested as an alternative to reduce the production cost (Rajaram and Varma 1990). It represents a significant resource of renewable biomass which can be utilized as a substrate for the preparation of many useful products such as fuels, solvents and chemicals. For most of the bioconversion processes, xylan needs to be converted to xylose or xylo**-**oligosaccharides. Primary intention of any pretreatment is to change or depolymerize the structural and compositional inconvenience to hydrolysis and subsequent degradation takes place in order to increase the digestibility, enhance the rate of enzyme hydrolysis and increase yield of prospective products (Mosier et al. 2005).

Alkaline pretreatment is one of the best choices that has several benefits compared to other pretreatment processes including operation cost reduction, less degradation of holocellulose and formation of intermediate inhibitors for downstream processing (Dhillon et al. 2000). The principle of alkaline pretreatment mechanism is the degradation of ester bonds between the lignin structure and cleavage of glycosidic linkages in the lignocellulosic cell wall carbon matrix, which indicates the alteration of lignin structure and depolymerization of the lignin**-**hemicellulose complex, swelling and partial decrystallization of cellulose (Sun and Cheng 2002).

Microbial xylanases are useful in many biotechnological industrial processes, such as pre**-**bleaching of kraft pulp (Bocchini et al. 2003), improving the digestibility of animal feed (Bedford and Classen 1992), juice clarification, and degumming of vegetal fibers such as jute, ramie and hemp (Kapoor et al. 2001). Xylanases are produced by a wide range of bacteria and fungi, including aerobes, anaerobes, mesophiles, thermopiles and extremophiles. Aerobic bacteria and fungi generally produce extracellular xylanases. The attack of the substrates is random and the bonds to be hydrolysed depend on the nature of substrate. During hydrolysis of xylan and arabinoxylan, the main products formed are xylo-oligosaccharides (Wong et al. 1988). Its complete hydrolysis is important in order to obtain, in higher yields, monosaccharides like d-xylose and l-arabinose are important, which could find applications in the food and fuel industries (Kim and Oh 2003). Enzymes from fungi such as the *Trichoderma* sp. deserve the most attention. *Trichoderma* sp. including *T. reesei, T. harzianum* and *T. viride* are well known as excellent producers of both xylanolytic and cellulolytic enzymes (Wong et al. 1988). Generally enzymes are produced by both SSF (solid state fermentation) and SmF (submerged fermentation), but SSF is natural like composting and ensiling.

The objective of the paper was to study the influence of “energy value” pretreated and untreated agro**-**waste substrates in different combinations on the improvement of the xylanase production by employing the *Trichoderma* sp. in SSF. The performance of the produced xylanase was also tested in the hydrolysis of pretreated natural biomass materials.

## Materials and methods

### Microorganism and culture media

Fifty fungal cultures were isolated and purified from sorghum litter collected from local agro-fields and maintained on sterile potato dextrose agar (PDA) slants at 4 °C and used as stock cultures. The isolates were identified on the basis of cultural characteristics and morphological features (Frazier and Westhoff 2008).

### Inoculum preparation

A 100 mL of the broth containing (g/L): glucose, 10.0; KH_2_PO_4,_ 2.0; MgSO_4_·7H_2_0, 0.5; CaCl_2,_ 0.2; NH_4_Cl, 0.10 and thiamine, 0.001 taken in 250 mL conical flask was sterilized at 121 °C for 15 min, cooled to room temperature, inoculated with *T*. *koeningi* spore suspension and incubated at room temperature on a rotary shaker (120 rev min^−1^) for 4 days to get a mycelial suspension as inoculum.

### Preparation and analysis of agro-waste

The substrates wheat straw (WS), rice straw (RS), mustard straw (MS), cotton straw (CS), sorghum straw (SS) and corn cobs (CC) collected from the local agro**-**fields were washed with distilled water twice and dried at 70 °C for 12 h in an oven. The oven**-**dried substrates were milled and passed through 0.5 mm mesh and were collected in the polythene bags for further use. The substrates composition (cellulose, hemicellulose and lignin) was determined by the AOAC method (2005). Neutral detergent fiber (NDF) is the unit of measurement for the cellulose, hemicellulose and lignin content and represents most of the fiber or cell wall fractions in biomass. Acid detergent fiber (ADF) was determined sequentially using the residue left after NDF determination. Lignin content was determined as acid detergent lignin (ADL). The hemicellulose content was determined by subtracting ADF from NDF (Jung and Vogel 1992).

### Pretreatment of agro**-**waste

The air dried and milled samples (WS, RS, MS, CS, SS and CC) were used as raw material. The samples were then pretreated with 1.0, 1.5 and 2.0 % (w/w) of NaOH for 90 h at 90 °C and washed with distilled water till the washed water reaches pH 7.0 and dried for 12 h at 80 °C. The pretreated agricultural substrate composition was determined by the AOAC method (2005). The reducing sugars released during the hydrolysis were estimated by using 3,5-dinitrosalicylic acid (DNS) method (Miller 1959). The pentoses (xylose and arabinose) were estimated by the method of Khabarov et al. (2006).

### Fruit peel powder preparation

Oven dried (at 70 °C for 7 h) fruit peels of mausambi (MUP), pineapple (PP), mango (MP) and banana (BP) were collected, ground and milled into 0.5 mm mesh to make into powdery form. The collected peel powder was stored in polythene bags. The moisture, ash, crude protein, cellulose, hemicelluloses and lignin were determined by the procedure of AOAC method (2005). Total carbohydrate content was determined by the method of Dubois et al. (1956).

### Reducing sugars estimation

This was carried out by using 3,5-dinitrosalicylic acid (DNS) method (Miller 1959). Sample (1 mL) was mixed with DNS (3 mL) and boiled for 10 min. The optical density (OD) values were checked at 540 nm to measure the colour intensity. The actual values were obtained by extrapolating with a standard graph of glucose or xylose.

### Total sugars estimation

To measure the total sugars in the hydrolysates, the phenol sulphuric acid method (Dubois et al. 1956) was used. Sample (1 mL) was mixed with 5 % phenol (1 mL) and 96 % H_2_SO_4_ (5 mL). The above mixture was kept for 10–15 min at room temperature. The OD values were checked at 470 nm to measure the colour intensity. The amount of sugars present in the sample solution was calculated using a standard graph. The absorbance corresponds to 0.1 mL of the test = *x* mg of glucose. 100 mL of the sample solution contains *x*/0.1 × 100 mg of glucose = % of total sugars present.

### Determination of protein

The protein content in the culture filtrate was estimated by Folin**–**Ciocalteu reagent using bovine serum albumin (BSA) as standard (Lowry et al. 1951).

### FT**-**IR analysis of pretreated corn cobs

The samples were prepared in the form of KBr (potassium bromide) discs, which were prepared by grinding 1 mg/100 mg KBr (FT**-**IR grade) in a vibratory ball mixture for 20 s. The 13 mm KBr discs were prepared under vacuum in a standard device under a pressure of 75 kN cm^−2^ for 2 min. The spectra resolution was measured as 4 cm^−1^ and scanning range of 500–4,000 cmK^−1^ by using PerkinElmer Spectrum 1 FT-IR.

### Enzyme production by SSF

The SSF experiments were carried out by using different agricultural wastes (WS, RS, MS, CS, SS and CC) along with combinations of fruit byproducts (MUP, PP, MP and BP) as solid material. 10 g of agro-residue (pretreated and untreated) and 5 g of byproducts was used in 500 mL Erlenmeyer flasks with basal medium of the following composition (g/L): KH_2_PO_4_, 1.6; (NH_4_)_2_SO_4_, 1.4; CaCl_2_·2H_2_O, 0.4; MgSO_4_·7H_2_O, 0.6; urea, 0.2; proteose peptone, 0.25; yeast extract, 0.2; moistened 60 % by supplementing with mineral salt solution [FeSO_4_·7H_2_O, 3; MnSO_4_·7H_2_O, 4.6; ZnSO_4_·7H_2_O, 3.34; CoCl_2_·2H_2_O, 1.5 mg/L (pH 7.0)]. The contents of the flasks were mixed properly and autoclaved at 121 °C for 15 min followed by cooling to room temperature and inoculated with 1 mL of *T. koeningi* (1.0 × 10^5^ spores per mL) spore suspension and incubated at room temperature for 7 days.

### Enzyme harvesting

After the completion of the SSF, the solid culture medium was extracted using 100 mL of 50 mM citrate buffer pH 5.0, under shaking at 150 rpm and 30 °C for 30 min, for three times. Then the aggregated contents were centrifuged at 4 °C for 15 min and the supernatant was collected and stored at 4 °C to prevent loss of enzymatic activities.

### Xylanase assay

Xylanase activity was measured by mixing 1.0 mL of 1 % (w/v) birch wood xylan (Sigma, USA) as substrate prepared in 50 mM sodium citrate buffer, pH 5.3 with 0.1 mL of diluted enzyme and the mixture was incubated at 50 °C for 5 min (Bailey et al. 1992). The reaction was terminated by the adding of 1.5 mL of 3,5-dinitrosalicylic acid (DNS) reagent and the mixture was boiled for 5 min (Miller 1959) followed by cooling. The colour intensity was measured at 540 nm using UV**–**Visible Spectrophotometer. The amount of reducing sugars was measured quantitatively by using xylose as standard. One unit of xylanase activity was defined as the amount of enzyme producing 1 µmol of equivalent to the xylose per min in the assay conditions.

### Cellulase assay

The FPase, CMCase (Reese and Mandles 1963) and β-glucosidase (Berghem and Petterson 1973) activities (cellulase) were determined using the standard methods.

### Optimization of pretreated biomass hydrolysis by xylanase

The pretreated biomass (3 g) was suspended at 2 % (w/v) consistency of 0.05 mM sodium citrate buffer, pH 5.3 taken in 50 mL conical flasks and reacted with different doses of *T. koeningi* xylanase like 12.0, 14.0 and 16.0 U for incubation periods of 2–8 h at 40 °C for determination of optimum conditions for hydrolysis.

### HPLC analysis of hydrolysed biomass

The Dionex DX-600 series liquid chromatograph (HPLC) was used for the quantification of samples. The HPLC system consisted of an AS50 auto injector, degassing module, GS50 gradient pump, LC30 chromatography oven and UV detector. Chromatographic separation was achieved using a 150 × 4.6 mm YMC carotenoid S**-**3 column (Milford). Gradient separations were carried out using aqueous 0.05 % (v/v) phosphoric acid (pH 2**–**2.3) and water: acetonitrile (10:90) as the A and B solvents, respectively. Additional parameters were: injection volume, 25 μL, column temperature, 30 °C, flow rate, 1 mL/min and detection wavelength at 210 nm. The xylose and xylo**-**oligosaccharides (xylobiose, xylotriose, xylotetraose, xylopentaose and xylohexaose) were eluted out with their gradients. Their retention time was compared with the external standards.

## Results and discussion

### FT-IR analysis of pretreated corn cob

**Fig. 1:**
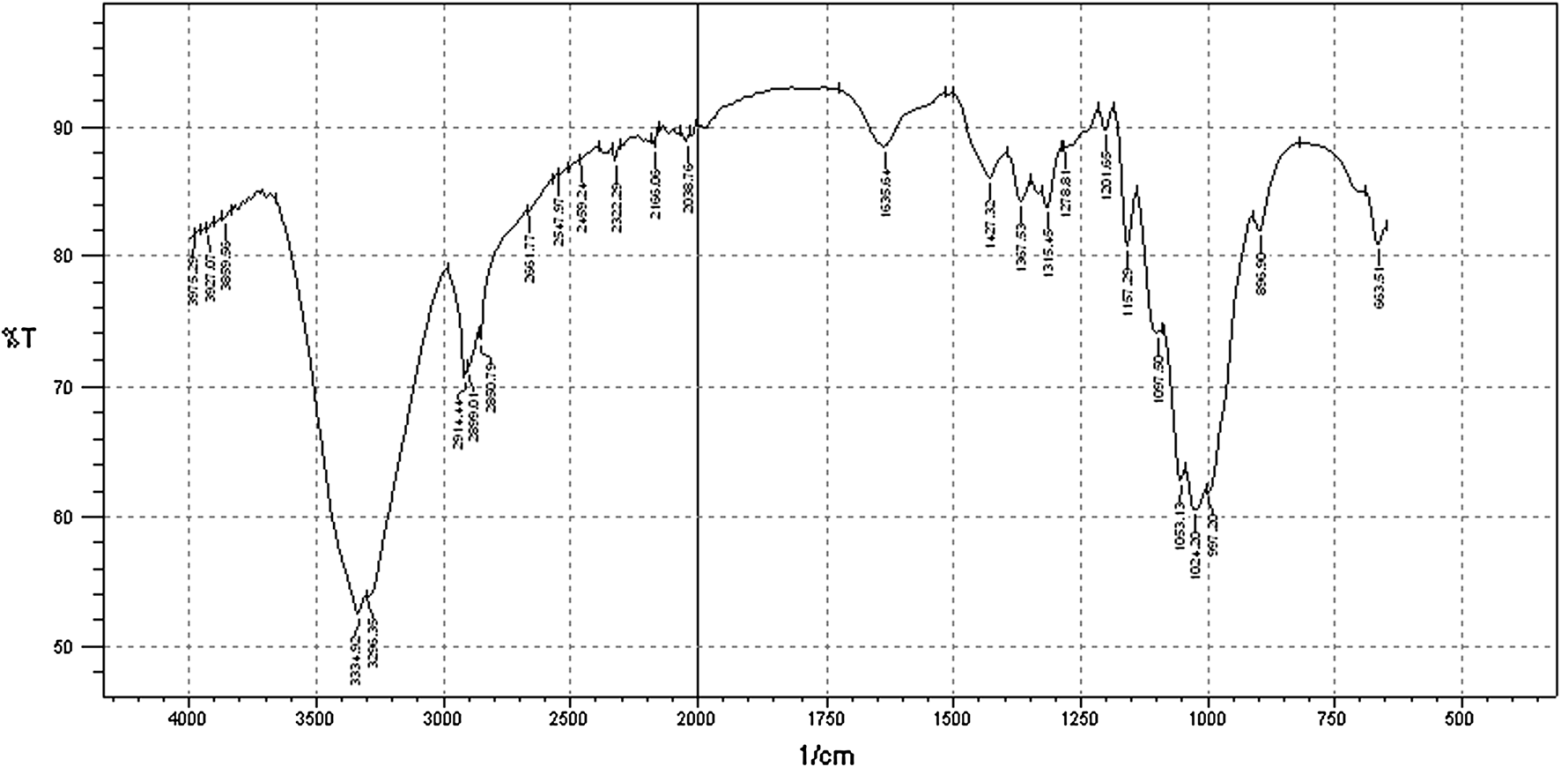
FTIR spectrum of alkali pretreated corn cob with a scanning range of 500–4,000 cm K^−1^

The Fig. 1 shows the FT-IR spectrum of extracted xylan using 2 % NaOH pretreatment. The spectrum is compared with the standard library of lignocellulose (Liang and Marchessault 1959). The pretreated corn cob spectrum band lengths were shown in the Table 1. It shows the dislocation or reduced the intensity of functional groups in the pretreated corn cobs, when compared to the standard lignocelluloses library. During the pretreatment some of the functional groups (1,635, 1,427 and 896 cm^−1^) were elevated and their peak assignment was in the Table 1. During NaOH pretreatment, the intra-molecular degradation of hemicellulose was represented by the decreased contents of functional groups and the disappearance of some bands in the pretreated spectrum compared with the standard spectrum. The peak at 1,252 cm^−1^ is indicative of C–O streaching of syringe units and the peak at 1,155 cm^−1^ is characterized by the C–O–C vibration in anomeric region of hemicellulose. The two bands were found disappeared, elucidating that the structure of hemicelluloses was changed after NaOH pretreatment, thus hemicellulose was degraded. Similarly the hemicellulose band appeared at 1,738 cm^−1^ for all original samples (Kumar et al. 2009). No hemicellulose band was observed after treatment of corn cobs, indicating that hemicellulose was depolymerised during the pretreatment process. The chemical composition analysis of biomass (Table 2) supports the FT**-**IR observations that the hemicellulose content of biomass decreased after pretreatment. The linear and branched (1-4)-β-xylans showed the main peak maximum at about 1,053 cm^−1^. It resembles that spectrum of pretreated corn cobs showed extraction of xylan and it is absent in the standard library. Lignin related bands in the spectrum were seen around 1,273, 1,518, 1,610 and 1,715 cm^−1^ (Kumar et al. 2009). The band at 1,518 cm^−1^ attributed to the C=C of lignin, was observed in lignocelluloses. This spectrum did not show the 1,518 cm^−1^ band after pretreatment. No detection of an absorption band at 1,715 cm^−1^, due to the C=O of the phenyl ester linkages between lignin and a hemicellulose had been cleaved by dilute alkali pretreatment. The typical adsorption of cellulose backbone at 1,635 cm^−1^, from the hydrogen bonded OH stretching at 3,000–4,000 cm^−1^, is due to the H bonded OH groups (at 3,334.92 cm^−1^) and the stretching frequency of the –OH as well as intramolecular and intermolecular hydrogen bonds (Richard 2002). The wide band between the 3,000 and 3,500 cm^−1^ was due to OH stretching vibrations of alcohols and phenols (Singh et al. 2005).

**Table 1:** Assignment of the FT-IR bands of functional groups in pretreated corn cob

Wavelength number (with dislocation)	Functional groups	Peak assignment	References
3,000–3,500 cm^−1^	O–H	Stretching of alcohol and could be due to presence of adsorbed water in the sample	Owen and Thomas (1989)
2,800–3,000 cm^−1^	C–H	Energy absorbed due to C–H bonds stretching of methyl and methylene (aliphatic and aromatic) groups	Ding et al. (2012)
2,000–2,350 cm^−1^	C≡C	Vibrations of C≡C bonds due to the weak bands	Wilson et al*. (*2000)
1,635 cm^−1^	C=O	Carbonyl stretching associated with aromatic rings	Shi and Li (2012)
1,427 cm^−1^	–CH_2_	Plane-bending vibrations	Schulz and Baranska (2007)
1,367–1,315 cm^−1^	C–O and C–H	Attributed to weak C–O stretching and C–H symmetric and asymmetric deformations	Yu et al. (2007) Sun et al. (2005)
1,207–1,157 cm^−1^	C–O	Due to weak C–O stretching and glycosidic linkage	Robert et al. (2005)
1,100–1,000 cm^−1^	–C–O, C=C and C–C–O	Vibrational stretching and can also be due to non-structural CHO bending.	Schulz and Baranska (2007)
896 cm^−1^	β-1-4	β-1-4 linkage	Robert et al. (2005)

**Table 2:** Composition of untreated and treated (with 2 % NaOH) biomass

	Cellulose	Lignin	Hemicellulose	Protein	Moisture	Ash	Total sugars	Reducing sugars
Substrate untreated								
W	35.9 ± 0.8	6.25 ± 0.2	28.7 ± 0.1	3.77 ± 0.4	6.0 ± 0.9	8.2 ± 0.7	ND	ND
R	39.7 ± 0.03	8.9 ± 0.8	23.5 ± 0.3	2.75 ± 0.8	8.3 ± 0.6	6.8 ± 1.4	ND	ND
M	27.8 ± 0.4	5.2 ± 0.6	17.8 ± 0.8	2.03 ± 0.3	4.1 ± 0.4	5.5 ± 1	ND	ND
C	51.2 ± 0.01	10.4 ± 0.6	24.7 ± 0.5	2.19 ± 0.7	5.5 ± 0.8	5.6 ± 0.7	ND	ND
S	23.5 ± 0.16	5.8 ± 0.9	21.1 ± 0.9	1.84 ± 0.5	5.8 ± 0.2	4.3 ± 0.4	ND	ND
CC	42.8 ± 0.06	5.9 ± 0.5	37.2 ± 0.3	3.79 ± 0.2	7.0 ± 0.4	7.1 ± 0.2	ND	ND
Substrate treated								
W	39.3 ± 0.2	2.1 ± 0.1	19.4 ± 0.6	4.19 ± 0.2	4.1 ± 0.8	9.6 ± 0.9	0.67 ± 0.4	0.62 ± 0.7
R	42.2 ± 0.02	4.1 ± 0.9	16.5 ± 0.3	2.85 ± 0.5	5.0 ± 1.5	9.8 ± 0.4	0.48 ± 0.7	0.43 ± 0.3
M	30.2 ± 0.2	2.17 ± 0.2	10.7 ± 0.7	2.28 ± 0.9	1.8 ± 0.6	7.5 ± 0.2	0.57 ± 0.3	0.53 ± 0.6
C	61.7 ± 0.6	7.8 ± 0.4	15.6 ± 0.8	2.72 ± 0.7	3.3 ± 0.9	8.0 ± 1.5	0.77 ± 0.2	0.75 ± 0.4
S	26.8 ± 0.04	3.67 ± 0.7	11.1 ± 0.5	1.97 ± 0.6	3.7 ± 0.6	7.1 ± 0.3	0.45 ± 0.7	0.42 ± 0.8
CC	40.4 ± 0.1	2.56 ± 0.4	21.9 ± 0.3	4.17 ± 0.3	4.8 ± 1.4	9.8 ± 0.8	0.82 ± 0.9	0.78 ± 0.2

Composition of untreated and treated biomass in percentage (%)

Except moisture remaining all the composition of substrates were done based on dry weight basis %

Values are mean of two replicates

*W* wheat straw, *R* rice straw, *M* mustard straw, *C* cotton straw, *S* sorghum straw, *CC* corn cobs, *ND* not detected

### Composition of agro-residues and fruit peels

The cell wall components of agro-residue were different due to the variation in the chemical composition of agro-residues with plant species, tissue type and region within the cell wall and development stages of the cell wall (Carpita and Mc Cann 2000). The untreated *Toona ciliate* holocellulose and lignin was high: 72.56 and 21.45 %, when compared to pretreatment with 1 % NaOH, it having the holocellulose 62.68 and lignin 15.45 %. Similarly the *Pinus roxburghii* holocellulose (67.24 %) and lignin (26.40 %) was high in untreated and in pretreatment the holocellulose (58.54 %) and lignin (19.2 %) was low (Kaushal et al. 2012). For pretreated substrates, a reduction in percentage of lignin components was observed along with the depolymerization of holocellulose. To identify the suitability of substrate for xylanase production under SSF conditions, the chemical composition of substrates was determined under standard experimental conditions. In the present study, the composition of structural agro**-**residues contained higher levels of cellulose, hemicellulose and lignin in the corn cobs and lower levels in the sorghum straw. High levels of moisture in rice straw, ash in wheat straw, protein in the corn cobs, simultaneously low levels of moisture in cotton straw, ash and protein contents in the sorghum straw was observed. During the pretreatment, some amount of total sugars was lost. This was high in case of corn cobs and low in case of sorghum straw (Table 2). Compared with acid processes, alkaline processes have less sugar degradation. Sodium, potassium, calcium and ammonium hydroxide are suitable alkaline pretreatment agents. Among the four alkaline agents, sodium hydroxide has been the most studied (Soto et al. 1994).

The proximate compositions of fruit peels are shown in Table 3. From the data it was observed that hemicellulose content was present in a significant quantity in all fruit peels followed by cellulose and lignin. It is clear that there was a relatively low level of lignin, which makes the fruit peels amenable to the utilization of fungal strains. The protein content was high in the mango peel and low in the mausambi peel. Li et al. (2010) reported relatively high amount of hemicellulose, cellulose and lower value of a lignin in citrus peel. According to studies of Chen and Wyman (1991) the carbohydrates stimulated the growth of fungi; in the present study, pineapple peel contained high amount of total carbohydrates 29.19 ± 0.04 % (w/v) that served as growth of the fungi and production of xylanase in the medium. The total carbohydrate content is higher in pineapple peel and followed mango, banana and mausambi peel.

**Table 3:** Composition of fruit peels

Substrates	Cellulose	Hemicellulose	Lignin	Protein	Moisture	Ash	Total carbohydrates
Mausambi peel	14.96 ± 0.01	16.08 ± 0.02	8.89 ± 0.02	5.4 ± 0.03	5.3 ± 0.05	5.8 ± 0.01	14.24 ± 0.06
Pineapple peel	20.6 ± 0.04	27.23 ± 0.05	10.30 ± 0.04	8.7 ± 0.02	9.4 ± 0.02	3.9 ± 0.03	29.19 ± 0.04
Mango peel	13.2 ± 0.03	20.84 ± 0.06	7.73 ± 0.01	9.5 ± 0.05	7.3 ± 0.05	6.4 ± 0.05	20.73 ± 0.06
Banana peel	24.6 ± 0.02	13.46 ± 0.01	11.62 ± 0.04	6.02 ± 0.03	10.5 ± 0.03	4.2 ± 0.02	17.83 ± 0.02

Except moisture remaining all the composition of substrates were done based on dry weight basis %

Values are mean of two replicates

### Effect of pretreatment on xylanase production

Kleinert and Barth (2008) reported that chemical pretreatment of lignocelluloses causes swelling leading to an increase in internal surface area, decrease in the degree of polymerization, separation of structural bonds between lignin and carbohydrates. In the present study, the agro-residues were pretreated with different concentrations (1.0, 1.5 and 2.0 %) NaOH. The composition of plant cell mainly consisted of cellulose followed by hemicellulose (xylan), greater the hemicellulose more the depolymerization was occurred which was freely available to the growth of fungal strain and hence greater enzyme production. The 2 % NaOH pretreated agro-residues led to maximum xylanase enzyme production. Increased concentration of NaOH beyond 2 % decreased the enzyme production. The pretreatment caused the removal of lignin effectively, while at the same time loosened the structure of lignin and hemicellulose. The internal structure of hemicellulose breakdown improved the porosity characteristic of substrate. A significant difference in enzyme activity and protein was observed with the change in alkali concentration and reached maximum 1,267.3 ± 0.6 IU/g and 4.3 ± 0.2 mg/g, respectively in corn cobs by *T. koeningi* (Table 4). Most strains of the *Trichoderma sp.* are notable producers of extracellular enzymes including important plant cell-wall hydrolyzing enzymes such as xylanases (Biely 1985).

**Table 4:** Production of xylanase by untreated and pretreated biomass with different concentrations of NaOH

Substrate (g)	Xylanase (IU/g)				Protein (mg/g)			
	Untreated	Pretreated			Untreated	Pretreated		
		1.0	1.5	2.0		1.0	1.5	2.0
W	580 ± 0.8	622.4 ± 0.7	678.2 ± 0.2	734.6 ± 0.5	3.1 ± 0.1	3.7 ± 0.4	3.8 ± 0.2	4.2 ± 0.6
R	217.7 ± 0.5	222.6 ± 0.4	243.7 ± 0.4	278.6 ± 0.8	2.9 ± 0.6	1.1 ± 0.5	1.4 ± 0.7	1.9 ± 0.4
M	156.3 ± 0.9	148.7 ± 0.8	156.5 ± 0.9	169.7 ± 0.4	2.1 ± 0.2	0.9 ± 0.2	0.9 ± 0.3	1.0 ± 0.5
C	189.8 ± 0.6	196.5 ± 0.3	199.8 ± 0.4	217.7 ± 0.2	2.8 ± 0.1	1.0 ± 0.7	1.1 ± 0.6	1.2 ± 0.3
S	169.9 ± 0.7	170.6 ± 0.7	182.9 ± 0.8	198.7 ± 0.4	1.9 ± 0.7	0.9 ± 0.4	1.0 ± 0.1	1.1 ± 0.8
CC	810.7 ± 0.1	745.7 ± 0.2	778.8 ± 0.7	1267.3 ± 0.6	3.7 ± 0.4	4.3 ± 0.2	4.5 ± 0.3	4.5 ± 0.2
W+MUP	520.6 ± 0.4	479.2 ± 0.9	538.6 ± 0.6	578.8 ± 0.1	2.6 ± 0.8	2.4 ± 0.9	2.7 ± 0.2	3.1 ± 0.6
R+MUP	151.5 ± 0.6	172.8 ± 0.4	189.7 ± 0.9	196.9 ± 0.8	1.7 ± 0.3	0.9 ± 0.4	1.0 ± 0.3	1.4 ± 0.4
M+MUP	131.2 ± 0.2	136.7 ± 0.1	142.7 ± 1.2	157.8 ± 0.4	1.2 ± 0.5	0.7 ± 0.4	0.8 ± 0.7	0.9 ± 0.5
C+MUP	159.2 ± 0.8	147.7 ± 0.4	157.4 ± 0.7	168.7 ± 0.2	2.4 ± 0.9	0.8 ± 0.4	0.9 ± 0.4	1.7 ± 0.3
S+MUP	137.8 ± 0.5	145.6 ± 0.7	154.8 ± 0.5	167.4 ± 0.7	1.7 ± 0.4	0.8 ± 0.3	0.9 ± 0.5	1.4 ± 0.2
CC+MUP	645.4 ± 0.4	639.1 ± 0.8	665.4 ± 0.8	721.4 ± 0.6	2.7 ± 0.1	3.9 ± 0.6	4.1 ± 0.7	4.4 ± 0.3
W+PP	653.8 ± 0.3	678.6 ± 0.1	697.9 ± 0.5	733.8 ± 0.4	3.8 ± 0.3	3.2 ± 0.4	3.6 ± 0.1	3.8 ± 0.9
R+PP	241.0 ± 0.6	229.8 ± 0.9	241.0 ± 0.8	273.8 ± 0.9	2.9 ± 0.1	2.0 ± 0.8	2.1 ± 0.9	2.8 ± 0.2
M+PP	189.4 ± 0.5	206.8 ± 0.2	226.5 ± 0.6	248.8 ± 0.3	2.4 ± 0.3	1.9 ± 0.4	2.0 ± 0.6	2.3 ± 0.6
C+PP	251.6 ± 0.9	289.6 ± 0.7	309.8 ± 0.2	347.4 ± 0.1	3.1 ± 0.6	2.6 ± 0.7	2.9 ± 0.4	3.1 ± 0.5
S+PP	237.4 ± 0.3	269.7 ± 0.9	293.6 ± 0.4	322.6 ± 0.3	2.2 ± 0.4	2.2 ± 0.2	2.4 ± 0.4	2.9 ± 0.7
CC+PP	1,347.2 ± 0.7	1,586.9 ± 0.6	1,795.4 ± 0.4	2,869.8 ± 0.4	4.9 ± 0.1	5.3 ± 0.6	5.8 ± 0.5	7.6 ± 0.2
W+MP	587.8 ± 0.5	616.9 ± 0.4	637.5 ± 0.7	686.9 ± 0.2	3.3 ± 0.4	3.5 ± 0.8	3.7 ± 0.6	3.8 ± 0.4
R+MP	262.4 ± 0.9	256.8 ± 0.8	279.7 ± 1.5	295.8 ± 0.7	2.4 ± 0.3	2.1 ± 0.5	2.2 ± 0.7	2.6 ± 0.3
M+MP	151.8 ± 0.2	144.6 ± 0.4	161.3 ± 0.4	193.8 ± 0.4	1.9 ± 0.5	0.7 ± 0.1	0.9 ± 0.2	1.0 ± 0.3
C+MP	209.1 ± 0.4	195.9 ± 0.7	217 ± 0.9	243.3 ± 0.8	2.3 ± 0.7	1.1 ± 0.4	1.9 ± 0.5	1.5 ± 0.5
S+MP	183.4 ± 0.6	183.4 ± 0.6	198.9 ± 1.6	223.8 ± 0.6	1.7 ± 0.3	1.0 ± 0.1	1.1 ± 0.6	1.3 ± 0.1
CC+MP	709.2 ± 0.6	730.6 ± 0.3	768.6 ± 0.8	794.7 ± 0.9	2.6 ± 0.1	3.7 ± 0.4	3.9 ± 0.3	4.1 ± 0.6
W+BP	542.1 ± 0.9	589.8 ± 0.1	609.6 ± 0.4	647.3 ± 0.2	2.9 ± 0.5	2.9 ± 0.6	3.1 ± 0.3	3.2 ± 0.7
R+BP	188.7 ± 0.2	198.7 ± 0.4	221.6 ± 0.7	267.5 ± 0.8	1.8 ± 0.3	1.2 ± 0.3	1.3 ± 0.7	1.7 ± 0.2
M+BP	131.8 ± 0.7	152.7 ± 0.9	171.5 ± 0.1	191.4 ± 0.4	1.5 ± 0.5	0.8 ± 0.5	0.9 ± 0.8	1.2 ± 0.5
C+BP	183.6 ± 0.3	196.7 ± 0.6	219.4 ± 0.7	238.7 ± 0.2	2.6 ± 0.7	1.1 ± 0.1	1.2 ± 0.1	1.9 ± 0.3
S+BP	163.9 ± 0.6	184.8 ± 0.2	198.8 ± 0.4	231.1 ± 0.5	1.2 ± 0.3	1.2 ± 0.8	1.3 ± 0.2	1.5 ± 0.6
CC+BP	644.7 ± 0.7	687.6 ± 0.2	710.7 ± 0.8	745.1 ± 0.6	2.3 ± 0.4	3.3 ± 0.6	3.5 ± 0.5	3.6 ± 0.4

*W* Wheat straw, *R* rice straw, *M* mustard straw, *C* cotton straw, *S* sorghum straw, *CC* corn cobs, *MUP* mausambi peel, *PP* pineapple peel, *MP* mango peel, *BP* banana peel

### Enhancing the xylanase production using fruit peels powder

**Fig. 2:**
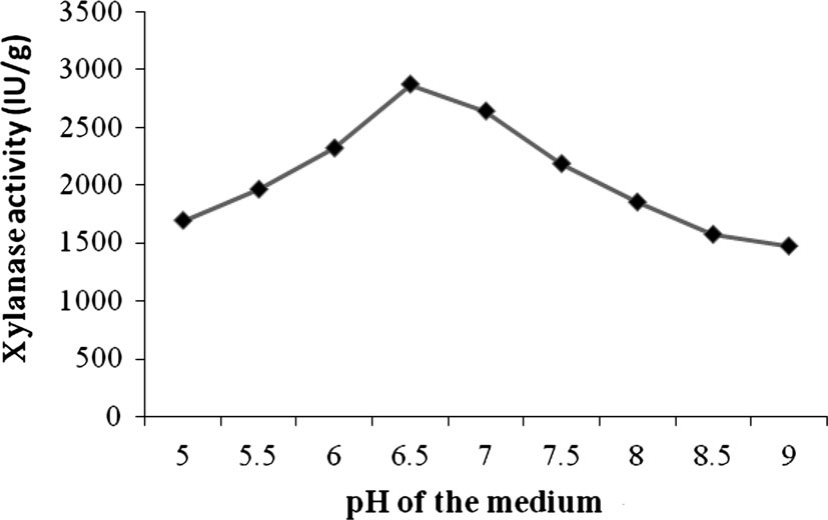
Effect of pH on xylanase production in SSF

**Fig. 3:**
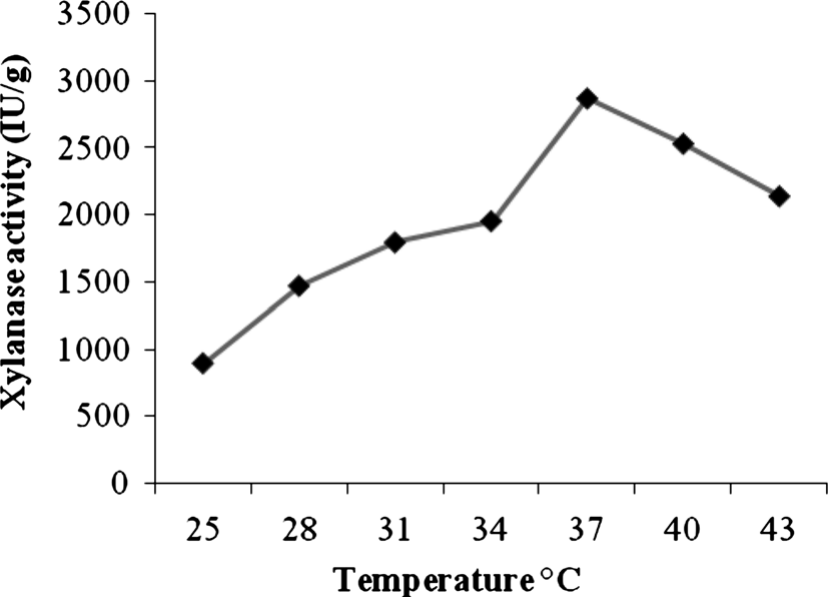
Effect of temperature on xylanase production in SSF

Currently, it could be considered as low cost strategy is the use of byproducts of industries as additional carbon source for the production of the enzymes. Villas-Boas et al. (2000) have used apple pomace for the production of xylanase by *Candida utilis*, whose cost was found to be comparatively low. Reddy et al. (2003) studied the xylanase production from *Pleurotus sp.*, using banana wastes. Large scale production of xylanase using pure xylan is uneconomical because of its cost. Hence cheap fruit peels were used as source of carbon material. Different locally available fruit- peels were collected and supplemented with various agro-residues in SSF. Among all the fruit peels, the pineapple peel powder combined with corn cobs enhanced significantly xylanase 2,869.8 ± 0.4 IU/g and protein 7.6 ± 0.2 mg/g, respectively (Table 4). Xylanase showed with maximal activity at pH 6.5, there was less xylanase at pH 5, and the activity was declined with increase in pH of 7.0–9.0 (Fig. 2). Gomes et al. (1992) reported that the maximal xylanase production obtained SSF conditions with pH 7.0 which was near to the present result. The xylanase activity was low at 25 °C declined after 37 °C and attained maximum activity at 37 °C. The results of the present study was near to the to the results of Gupta et al. (2009) found 30 °C as optimum temperature for maximum xylanase production with wheat bran by *Fusarium solani* F7 respectively (Fig. 3). The loss of enzyme activity at high temperature is due to the denaturation and conformational changes.

### Hydrolysis of pretreated biomass by xylanase

**Fig. 4:**
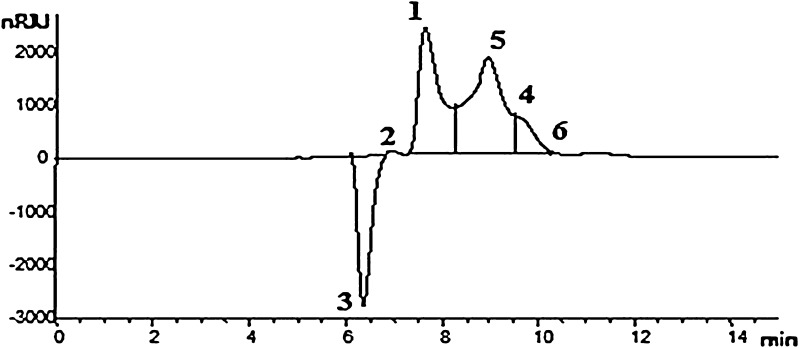
The HPLC chromatogram showing the profile of hydrolysed products from corn cobs (*Peak 1* xylose, *Peak 2* xylobiose, *Peak 3* xylotriose, *Peak 4* xylotetraose, *Peak 5* xylopentaose, *Peak 6* xylohexaose)

Kambourova et al. (2006) performed xylanase from *Anoxybacillus flavithermus* BC treated oat spelt, birch wood and beech wood xylan as substrate and the main sugar was xylose. In this direction, the pretreated biomass was reacted with *T. koeningi* xylanase for different incubation periods (2, 4, 6 and 8 h) at 14 U enzyme dose and obtained the xylose and xylo-oligosaccharides. Figure 4 illustrates the xylo-oligosaccharides profile (Peak 1 = xylose; Peak 2 = xylobiose; Peak 3 = xylotriose, Peak 4 = xylotetraose, Peak 5 = xylopentaose, Peak 6 = xylohexaose) of pretreated corn cobs and Table 5 clearly shows the released hydrolysates of pretreated biomass quantitatively by HPLC. In the 4 and 6 h incubation maximum xylose, xylobiose xylotriose and xylohexaose was observed, but in the 6 h and beyond, there was constant amount of xylose due to insufficient or incomplete hydrolysis of substrate. In case of xylotetraose and xylopentaose, they increased in pretreated corn cobs from 4 to 6 h. Alkali pretreated wheat straw was saccharified using xylanase alone or mixture of FPase and β-glucosidase at 6 h increased the reducing sugars (Kumar et al. 2013). The use of agro**-**wastes in the production of enzymes such as xylanase and valuable products is cost-effective, at the same time reduces the environmental pollution due to the dumping of such wastes.

**Table 5:** Estimation of hydrolyzed products using HPLC from xylanase-treated biomass

Substrate (g)	Concentration of sugars (mg/mL) at different time (h) intervals											
	Xylose		Xylobiose		Xylotriose		Xylotetraose		Xylopentaose		Xylohexaose	
	4 h	6 h	4 h	6 h	4 h	6 h	4 h	6 h	4 h	6 h	4 h	6 h
W	3.2	3.9	1.3	2.4	0.6	2.1	0.9	1.4	1.9	2.4	1.9	1.1
R	1.5	2.7	1.4	2.1	0.5	0.9	0.2	1.2	1.9	2.4	1.2	0.8
M	1.2	2.2	1.2	1.7	0.8	1.4	0.3	0.6	1.3	1.7	0.6	0.3
C	0.6	1.6	0.9	1.2	0.5	1.3	0.4	0.5	0.9	1.1	0.7	0.2
S	0.4	1.1	0.4	0.7	0.1	0.9	0.3	1.0	1.6	1.9	1.8	0.7
CC	4.2	6.8	2.6	3.7	1.8	2.6	1.2	3.1	3.8	4.2	2.8	1.4

*W* Wheat straw, *R* rice straw, *M* mustard straw, *C* cotton straw, *S* sorghum straw, *CC* corn cobs

## Conclusion

The present work demonstrated that the alkaline pretreatment method could significantly alter the physico**-**chemical structure of the substrates. The pretreated substrate composition clearly states that the cellulose content was increased but hemicellulose and lignin contents were decreased. The FT-IR data shows the depolymerization of pretreated corn cobs, which was easily utilized by the fungus. The untreated agro**-**residue yielded lesser xylanase than the pretreated agro**-**residue. Besides, the pretreated substrates combined with the fruit peels enhanced the xylanase production by SSF. The isolated *T. koeningi* xylanase degraded the pretreated biomass and the resultant hydrolysed products were quantified using HPLC. The results are encouraging from the point of view of obtaining cheap source of xylanase from agro**-**residues and their application in converting hemicellulose to fermentable sugars for commercial application.
